# Prenatal Diagnosis and Prognostic Factors in Fetuses With Arthrogryposis Multiplex Congenita—A Systematic Review

**DOI:** 10.1002/jcu.23983

**Published:** 2025-03-28

**Authors:** Mario Brock Leao, Marianna Facchinetti Brock, Jorge Roberto Di Tommaso Leao, Gianpaolo Grisolia, Mario Lituania, Samantha Erin Neumann, Gabriele Tonni, Rodrigo Ruano

**Affiliations:** ^1^ University of South Florida Tampa Florida USA; ^2^ University of the Amazon State Manaus Brazil; ^3^ Department of Obstetrics and Gynecology Carlo Poma Hospital, AST Mantova Mantova Italy; ^4^ Preconceptional and Prenatal Pathophysiology, Department of Obstetrics and Gynecology, E.O. Ospedali Galliera Genoa Italy; ^5^ Department of Human Genetics University of Miami, Miller School of Medicine Miami Florida USA; ^6^ Prenatal Diagnostic Centre, Department of Obstetrics and Neonatology Istituto di Ricovero e Cura a Carattere Scientifico (IRCCS), AUSL Reggio Emilia Italy; ^7^ Division of Maternal‐Fetal Medicine, Department of Obstetrics, Gynecology & Reproductive Sciences University of Miami, Miller School of Medicine Miami Florida USA

**Keywords:** arthrogryposis multiplex congenita, outcomes, prenatal imaging, prognosis, ultrasound

## Abstract

Arthrogryposis Multiplex Congenita (AMC) is characterized by the presence of multiple joint contractures in the fetus' body. The diagnosis of AMC is complex and often missed prenatally. This comprehensive review of the literature published over the last 20 years, with an emphasis on the role of prenatal ultrasound in predicting postnatal outcomes in a fetus with AMC, showed that ultrasound is a crucial tool to diagnose and prognosticate this rare disease. Different clinical manifestations of AMC are associated with different outcomes. Postnatal prognosis depends on several factors. Future multicenter prospective studies are necessary to investigate the prognostic factors associated with AMC.

## Graphical abstract

Arthrogryposis Multiplex Congenita (AMC) is characterized by the presence of multiple joint contractures in the fetus' body. The diagnosis of arthrogryposis is complex and often missed prenatally. This comprehensive review of the literature published over the last 20 years, with an emphasis on the role of prenatal ultrasound in predicting postnatal outcomes in a fetus with AMC, showed that ultrasound is a crucial tool to diagnose and prognosticate the AMC. Different clinical manifestations of AMC are associated with different outcomes. Postnatal prognosis depends on several factors. Future multicenter prospective studies are necessary to investigate the prognostic factors associated with AMC.

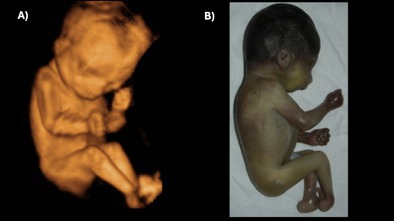

## 1 Introduction

Arthrogryposis, also referred to as Arthrogryposis Multiplex Congenita (AMC), is a disease characterized by joint contractures in at least two different sites of the fetus' body [1, 2]. The development of AMC is associated with decreased fetal movement or fetal akinesia [1, 2, 3]. It occurs in approximately 1 in every 3000–5000 births [4, 5]. Over 400 different conditions have been associated with AMC. Although this condition usually affects the limbs, it may also involve contractures in the neck, jaw, and spine at birth [1, 2, 4, 6]. AMC has a wide range of etiologies and associated anomalies. Postnatal prognosis varies considerably depending on different factors [7]. Anomalies such as opisthotonos, pterygia, scoliosis, hydrops, nuchal edema, and absent filling of stomach are usually associated with intrauterine fetal demise (IUFD) or neonatal death [7]. AMC is a variable disease with more than 400 genes implicated in etiology. Therefore, clinical prognosis and outcome are related to the underlying pathogenetic mechanism [8]. Intrinsic mechanisms and etiologies are considerably more diverse than extrinsic factors. This group of disorders usually stems from abnormalities in the brain, spinal cord, muscles, bones, tendons, joints, restrictive dermopathies, neuromuscular junctions, and peripheral nerves [8]. Due to the great variety of causes and clinical manifestations in AMC, there is a high variability also in postnatal outcomes, especially the group of intrinsic mechanisms, related to the complexity of treatment, involvement of developmental delays, and in some cases such as Patau, Edwards, and Pena‐Shokeir syndromes, association with lethality [1, 8, 9].

The present article is a comprehensive review of the etiology, classifications, prenatal findings, and predictors that may be useful in prenatal counseling to predict the postnatal outcome.

## 2 Materials and Methods

**FIGURE 1:**
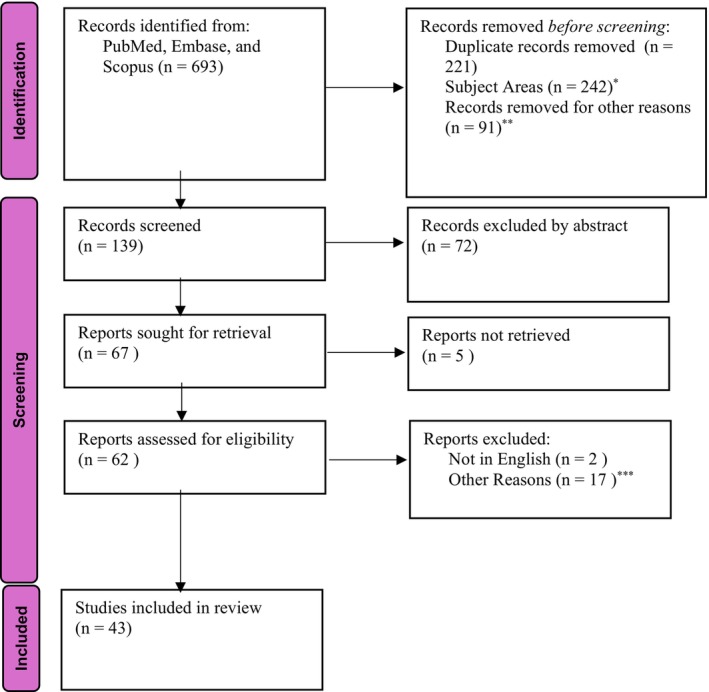
Flow diagram PRISMA 2020 of study selection. (From: Page MJ, McKenzie JE, Bossuyt PM, Boutron I, Hoffmann TC, Mulrow CD, et al. The PRISMA 2020 statement: An updated guideline for reporting systematic reviews. BMJ 2021;372:n71. *Records Excluded by title. **Records were outside of the period in which the review aimed to emphasize. ***Upon reading the full article, research focus and/or subject area of the report were outside of the scope of this review.

The primary aim of this current review is to analyze the recent literature on AMC with an emphasis on the role of prenatal ultrasound in predicting postnatal outcomes in a fetus with AMC. Furthermore, we have also investigated the different classifications, etiopathogenesis, genetic factors, prognosis, and postnatal management associated with the condition. An electronic search was conducted using three different databases, e.g., PubMed/Medline, EMBASE, and SCOPUS. Keywords searched were the following: arthrogryposis multiplex congenita, ultrasound, prenatal ultrasound, perinatal outcome, prognosis, postnatal treatment. The electronic search strategy is reported in Figure 1.

## 3 Results

A review of recent literature over the last twenty years (from January 2004 to December 2023) was conducted. Six hundred and ninety‐three articles were identified from the search engines, where 221 were duplicates (Figure 1) Two hundred and forty‐two articles were excluded based on title, and 91 articles were excluded because they were published outside of the 20‐year period this article aims to review. One hundred thirty‐nine records remained, where 72 were excluded based on the scope of the abstract, and of the 67 remaining, 5 articles were excluded due to not being accessible to the authors. The 62 remaining sources were assessed for eligibility, where 2 were not in English, and 17 were excluded based on research focus upon reading the full article. The remaining 43 studies were included in this review.

### 3.1 Etiology and Classification

Etiologies of AMC can be divided into 2 categories: extrinsic (extra fetal) and intrinsic (intra fetal) [2, 6, 8, 10]. Extrinsic factors include maternal factors such as infections, the use of alcohol/drugs, uterine abnormalities such as bicornuate uterus and septate uterus, uterine tumors, and multiple pregnancies which would result in the compression of the fetus [2, 6, 8]. The last two groups of etiologies are usually associated with deformation and more favorable outcomes; however, maternal factors are generally associated with poorer prognosis as they are also associated with central nervous system (CNS) abnormalities [8]. AMC mainly recognizes intrinsic mechanisms in its pathogenesis, such as amyoplasia, distal limb abnormalities, CNS disorders, connective tissue disorders, skeletal system disorders, and other genetic abnormalities [2, 4, 6, 10].

Since AMC can be caused by several different mechanisms, it can be challenging to define a single classification system that would serve every case of AMC [11].

**FIGURE 2:**
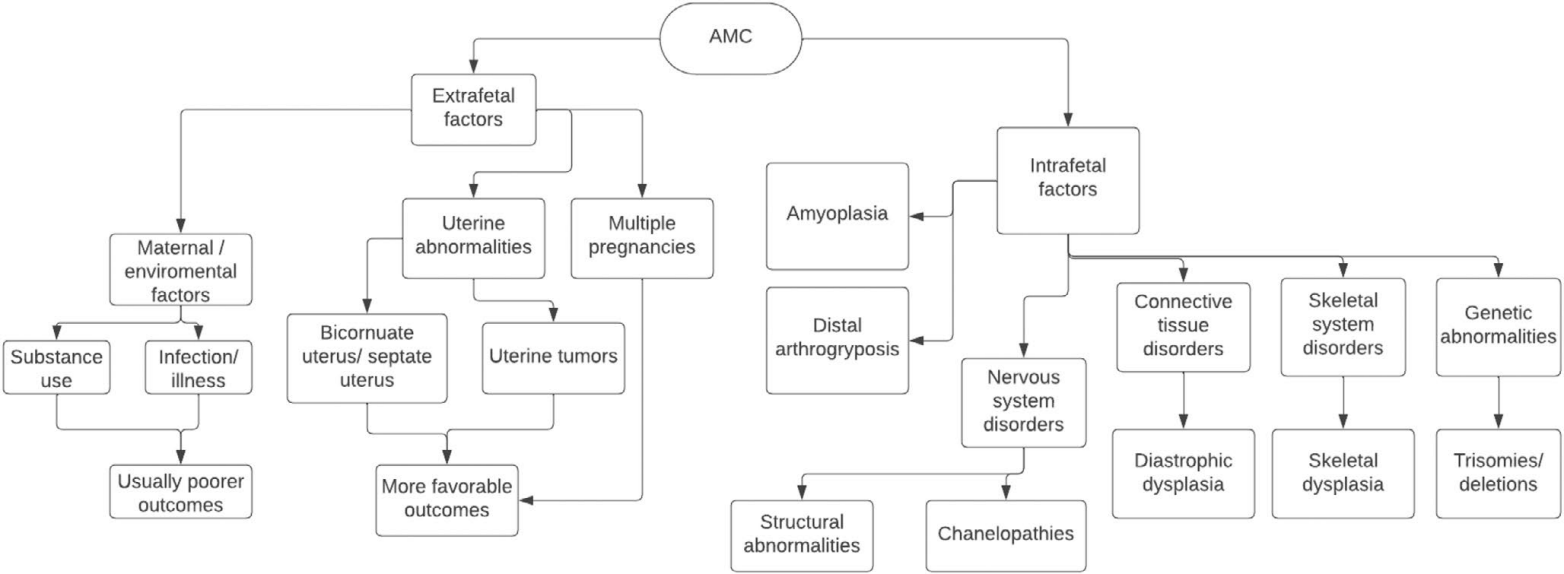
Classification of AMC by underlying causes of fetal akinesia. (Adapted from Langston and Chu, 2020).

Hall et al. [11] proposes four different systems of classification: classification by the underlying cause of fetal akinesia (intrinsic vs. extrinsic) (Figure 2), by area of involvement, by etiological process, and by the condition's cardinal features [11].

**FIGURE 3:**
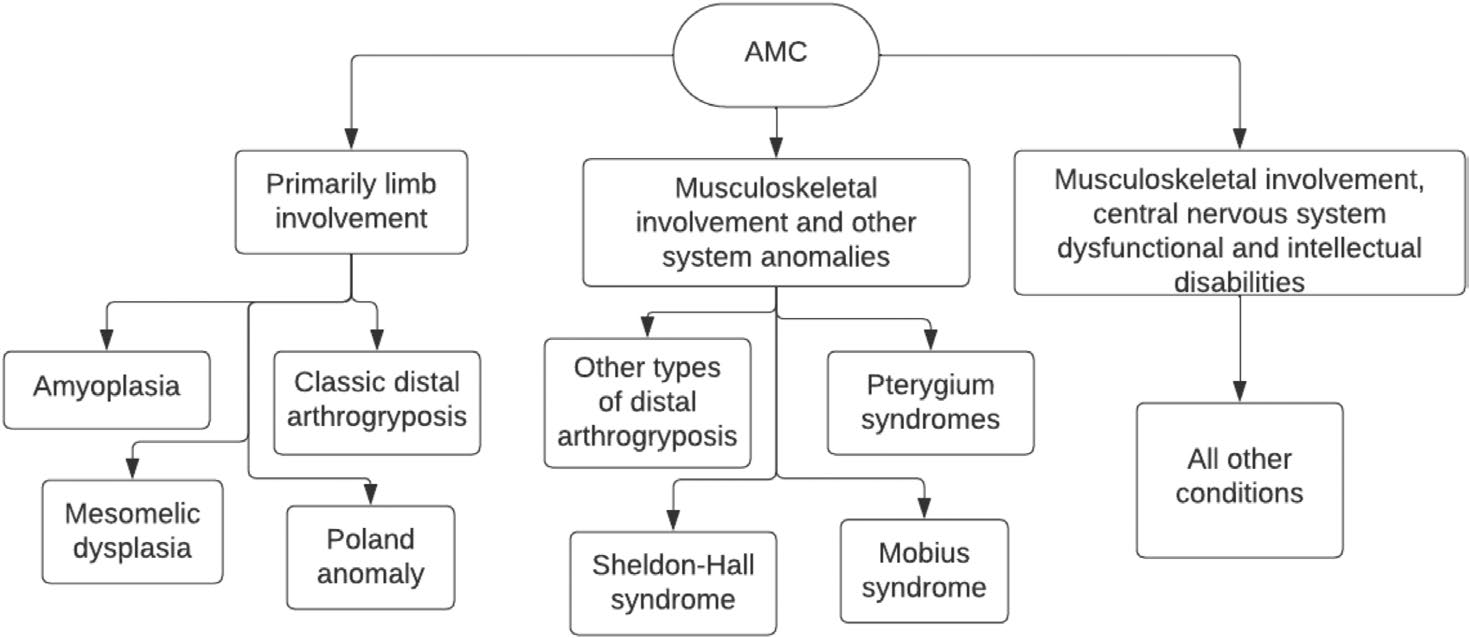
Clinical classification of AMC based on level of musculoskeletal involvement. (Adapted from Hall et al. [11]).

When classifying AMC by area of musculoskeletal involvement, it is divided into three groups (Figure 3).

Primarily limb Involvement, musculoskeletal involvement *plus* other system anomalies, and musculoskeletal involvement *plus* CNS dysfunction. The first group includes classic distal limb abnormalities, amyoplasia (the most common diagnosis to manifest with AMC), mesomelic dysplasia, Bruck syndrome, and Poland anomaly [2, 6, 8, 11]. The second group includes mostly the other types of distal limb abnormalities and pterygium syndromes. While the last group is the largest group, as it includes all the CNS disorders associated with AMC [11, 12].

**FIGURE 4:**
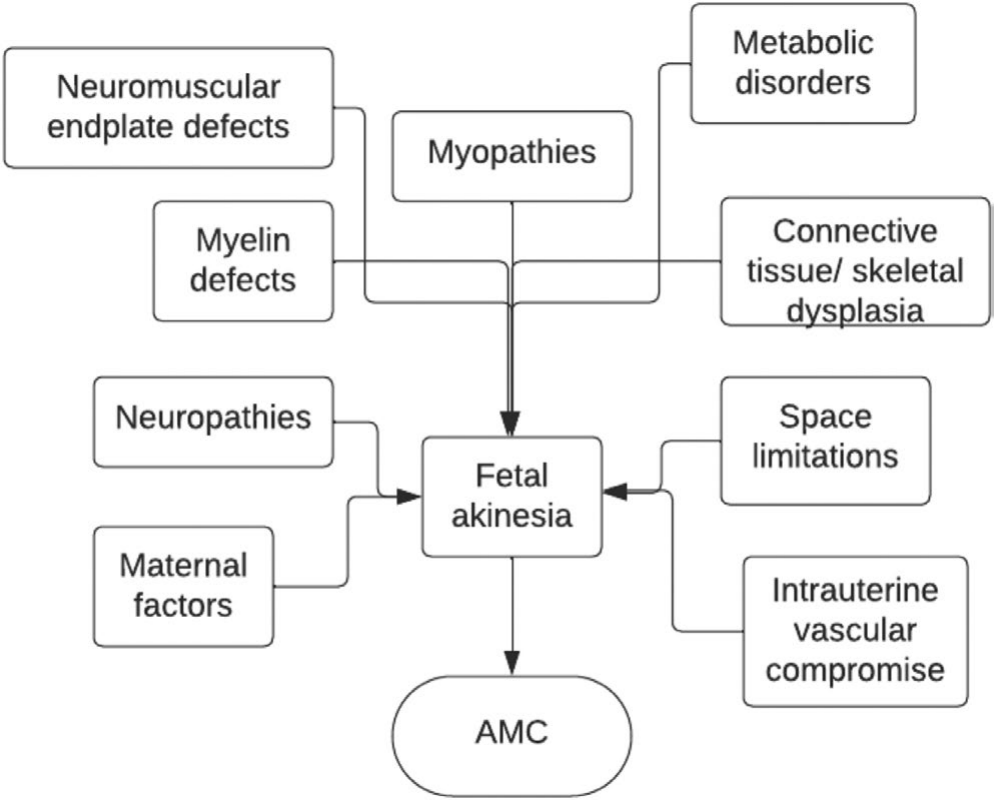
AMC classification by etiological process. (Adapted from Hall 2014).

The classification system by the etiological process is more complex as it includes 9 different categories: neuropathic defects, which include CNS, spinal cord, and peripheral nerve defects; myelin defects; neuromuscular endplate defects; myopathies; metabolic disorders; connective tissue disorders; skeletal dysplasias; space limitations; maternal factors such as infections or substance use; and intrauterine vascular compromise [12] (Figure 4).

**FIGURE 5:**
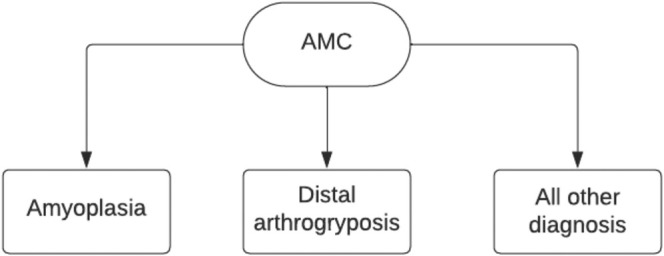
AMC classification by cardinal features. (Adapted from Hall et al. [11]).

Finally, the classification by cardinal features is most useful because it is effective in making the condition simple enough to explain the diagnosis to a family. This system is divided into three categories (Figure 5). Amyoplasia, distal limb abnormalities, and a “Rest” category, which would include maternal illness, other genetic conditions, fetal akinesia sequence, pterygium syndromes, teratogenic syndromes, and intellectual disabilities. Since amyoplasia and distal limb abnormalities make up over two thirds of the AMC cases, this system divides them into a separate category as it provides an opportunity for the physician to explain the condition in more detail [2, 8]. However, the third category is very broad, and as genetic discoveries emerge, it would benefit from further division.

### 3.2 Genetics

While not usually inherited from an affected parent, it is estimated that up to 61% of all AMC cases are due to genetic factors [1, 13, 14, 15]. Several types of inheritance have been associated with AMC, such as autosomal recessive (commonly associated with CNS disorders and severe fetal akinesia sequence), autosomal dominant (frequently associated with cases of distal limb abnormalities), *X*‐linked manners, maternal mitochondrial inheritance, and, in some cases, a mixture of genetic and environmental factors [1, 6, 13]. There are over 400 diseases associated with AMC; of those, more than half of them have a genetic basis [8, 11]. Since the diagnostic rates, even with whole genome sequencing, are not 100% and up to 32% of molecular diagnoses are attributed to newly discovered genes, it is not always possible to confirm the diagnosis or prognosis of AMC with a pathogenic genetic result [6, 14, 15]. Chorionic villus sampling or amniocentesis with array‐CGH is recommended when there is suspicion of AMC [1, 6]. However, when microarray does not provide a satisfactory explanation, further testing such as whole exome sequencing (WES) which should identify 60% of genetic causes of AMC [2, 14], or whole genome sequencing should be carried out [13, 16, 17].

Chromosomal abnormalities are detected in 90% of fetuses, most frequently trisomy 18 [4]. Another common genetic relationship is the association of AMC and scoliosis, with 227 out of 444 genes seen in AMC sharing features of these two conditions [17].

It is also important that if the pregnancy ends before a diagnosis and etiology conclusion are reached, an autopsy should be offered to further improve the understanding of the fetus' condition [1, 8].

### 3.3 Ultrasound Diagnosis

**FIGURE 6:**
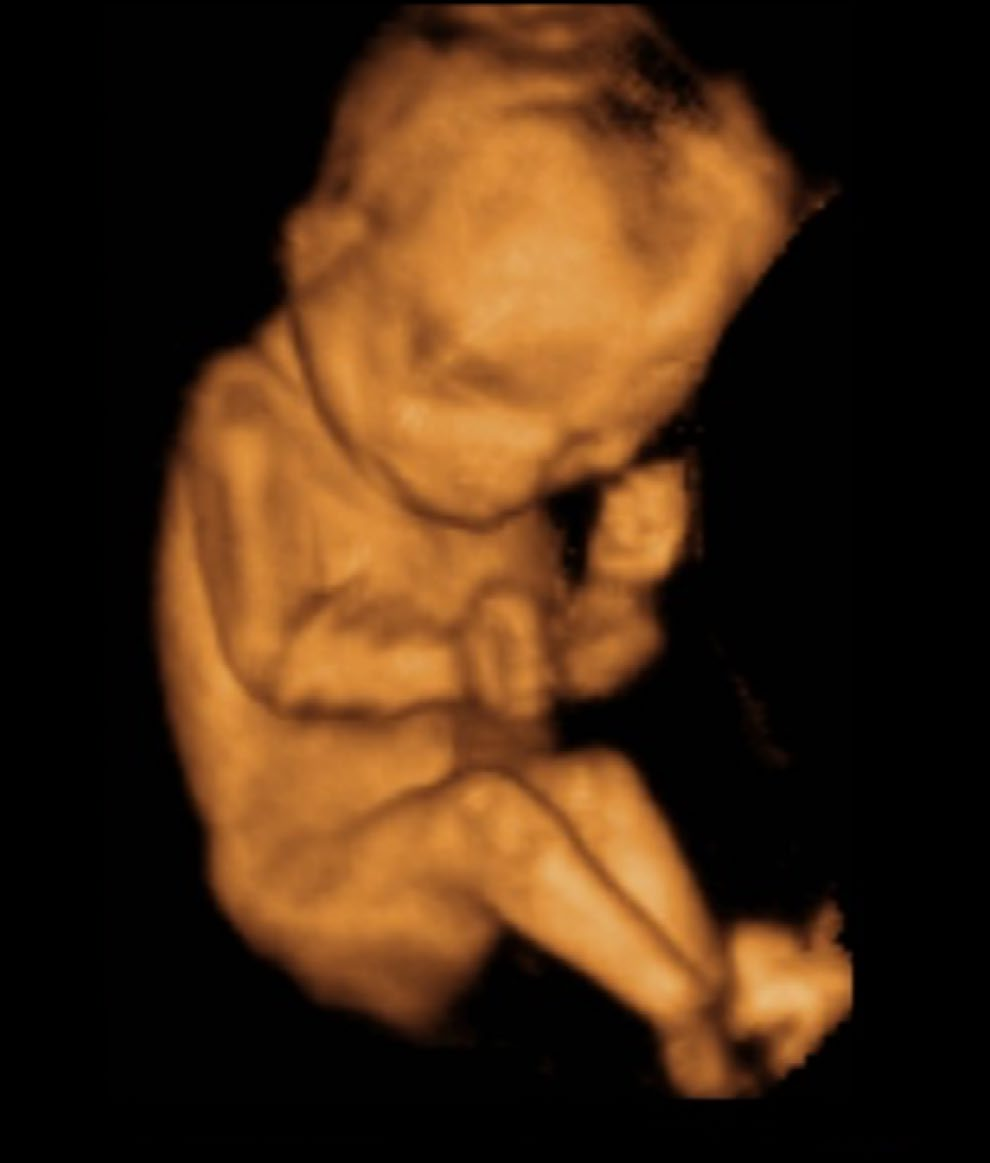
Three‐dimensional ultrasound imaging showing abnormal positions of the upper and lower extremities of a fetus with AMC.

Fetal movement starts in utero at 8 weeks of gestational age. Fetal joints, however, can only be visualized at 11 to 12 weeks gestation, and skeletal anomalies can only begin to be diagnosed at 14 weeks [8, 16, 18]. More recent techniques of ultrasound, such as 3D and 4D, have been proven useful in detecting symptoms of fetal akinesia and in the diagnosis of the Pena‐Shokeir phenotype [9, 19]. Even so, the majority of cases are only diagnosed after delivery, with recent studies showing an antenatal detection rate of 37% [1, 20, 21]. (Figure 6).

First‐trimester ultrasounds bear great importance in the diagnosis of AMC [19, 20, 22].

**FIGURE 7:**
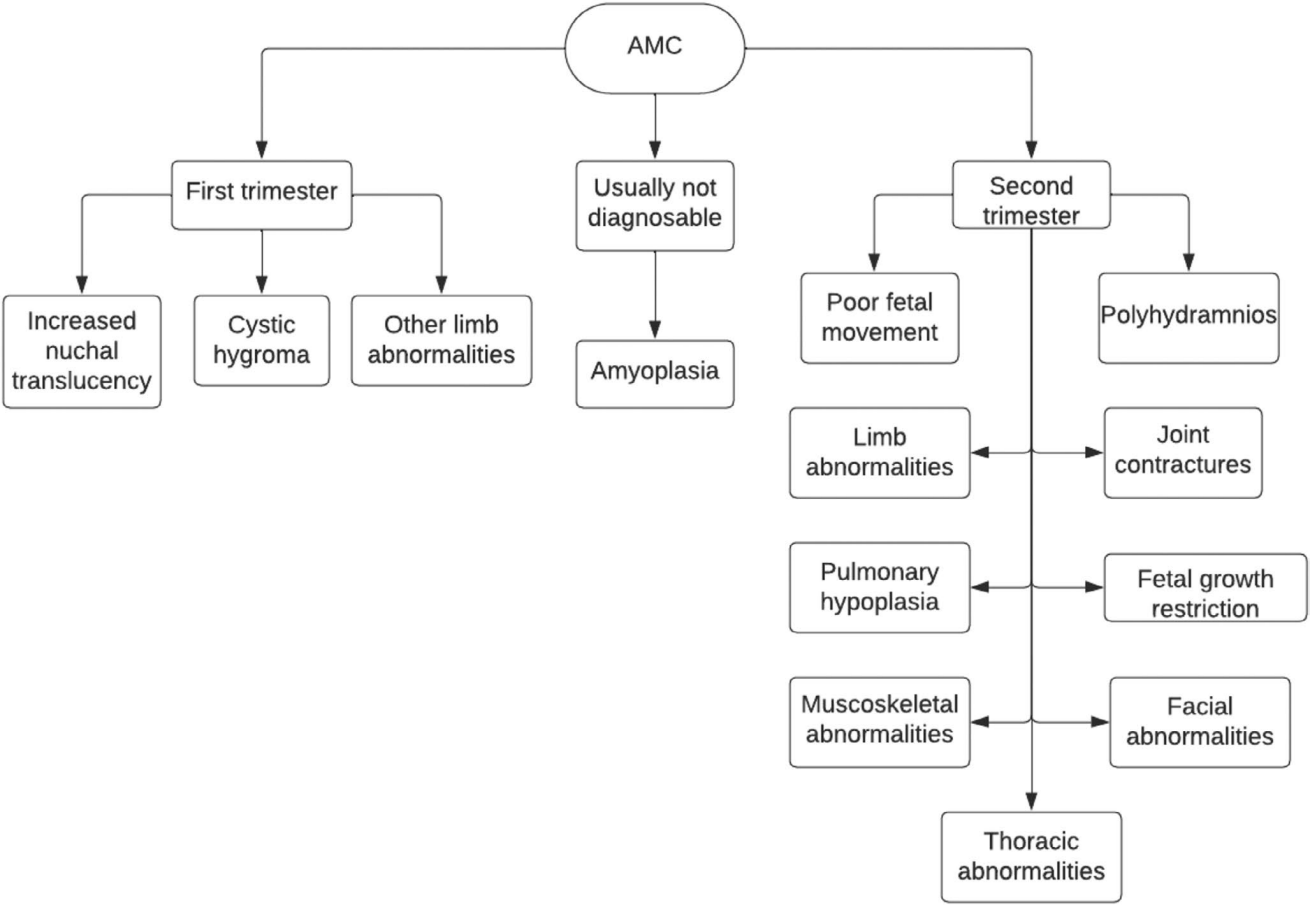
Flowchart of AMC diagnosis based on ultrasound in relation to gestational age.

At least 50% of malformations related to AMC can be diagnosed in the first trimester via ultrasound, and 10% of all AMC cases are identified in the first trimester [23]. Increased nuchal translucency, cystic hygroma, and other limb anomalies are some indicators of AMC that can be identified in the first trimester [8] (Figure 7).

The most common limb anomaly associated with AMC is clubfoot, which is not considered rare and is estimated to occur in 1/200 pregnancies. Clubfoot has been reported to be diagnosed in the first trimester and is estimated to occur in 83% of cases of AMC [18, 21]. Approximately 64% of cases where limb anomalies are present are diagnosed in the first trimester [24].

While first‐trimester ultrasounds are important, most cases of AMC are only diagnosed in the second trimester [13, 20]. Second‐trimester ultrasounds can properly visualize the quality of fetal movement, joint positioning, lung size, muscle growth, bone growth, as well as major organ anomalies [18, 22] (Figure 7).

All AMC cases with primarily limb involvement and some associated anomalies, such as chondrodysplasias, can be diagnosed via ultrasound in the second trimester as long as visualization of the fetus is not suboptimal [23]. Some common findings in the diagnosis of AMC in the second trimester include clenched hands (often associated with trisomy 18), joint contractures, abnormal extension, flexion, or position of the limbs, decreased fetal movements, and polyhydramnios (often due to decreased fetal swallowing) [8]. Secondary anomalies like pulmonary hypoplasia can be detected by assessing a narrow thoracic cage with consequent small thoracic circumference [25]. Other associated anomalies can include short umbilical cord, brain malformations, cleft lip/cleft palate (usually associated with trisomy 13), retrognathia/micrognathia, large cardiac size/small thorax, congenital diaphragmatic hernia, gastroschisis, scoliosis, pterygia, hydrops, intrauterine growth restriction, decreased muscle bulk, fractures, nonvertex presentation, absence of fetal stomach bubble, and hypoechogenicity of fetal long bones [8, 26]. Some of those abnormalities together (micrognathia, cleft palate, hypertelorism, fetal growth restriction, pulmonary hypoplasia, and mildly shortened long bones) make what is called the Pena‐Shokeir phenotype, estimated to occur in 1 every 12 000 births [27, 28].

Most types of AMC are diagnosed through ultrasound in the second trimester [13]. Amyoplasia is identifiable prenatally, but it has been shown to be difficult and requires an experienced sonographer to diagnose [6, 13]. AMC with amyoplasia and multiple contractures is not diagnosed until delivery in 75% of the cases [23]. This is suspected to happen because fetal movement is not routinely studied in ultrasound examinations and diagnosis often relies on reports from the mother [13]. A simple 15‐min systematic motor assessment has been shown to improve the rates of diagnosis in fetuses with AMC [29]. A complementary approach to the diagnosis of AMC can be successfully done using fetal magnetic resonance imaging (MRI) [30, 31]. Since this modality provides a shine‐through effect to other organs, it can be an alternative to 3D ultrasound in diagnosing fetal movement disorders, including limb anomalies [30].

Finally, in some cases, AMC may only be apparent in the third trimester [32]. The most common findings of fetuses with AMC in the third trimester are polyhydramnios, fetal talipes, pleural effusions, micrognathia, hydronephrosis, ventriculomegaly, and collapsed stomach [32].

Overall, the most common prenatal findings reported to be associated with AMC are clubfoot (83% of the cases), abnormal facial profile (65% of the cases), CNS abnormalities (62% of the cases), clenched hand, elbow contracture (both in 51% of the cases), decreased fetal movement, polyhydramnios, increased nuchal translucency (all in 50% of the cases), knee contracture (46% of the cases), and fetal hydrops (35% of the cases) [21, 33].

### 3.4 Outcomes, Postnatal Management, and Prognosis

Due to the extremely diverse nature of the etiology of AMC, there are several possible outcomes associated with the condition [23, 34]. Outcomes range from physical compromise with normal intellect to perinatal lethal [23, 34]. Besides other associated lethal conditions such as trisomy of 13 or 18 or the Pena‐Shokeir phenotype, some anomalies that can accompany AMC also show a high correlation with lethality such as opisthotonos, pterygium, and scoliosis [7, 9]. Hydrops, nuchal edema, absent filling of the stomach, and knee flexion joints show a better intrauterine outcome although they are heavily associated with neonatal death [7]. Thorax hypoplasia, pes equinovarus, and rocker bottom feet were associated with a more favorable post‐natal prognosis as most fetuses that survived birth tend to survive the neonatal period [7].

Some extrinsic mechanisms also heavily influence the outcome of pregnancy affected by AMC [8]. Maternal substance abuse and infections such as Zika virus infection in the presence of AMC were associated with an increased risk of fetal demise since those tend to compromise the CNS and likely will result in CNS abnormalities [35]. Patients that suffered from Zika virus infection associated with AMC were compared to patients that suffered just from the viral infection in pregnancy, and a 13‐fold risk of mortality was observed with an odds ratio of 13.11 and a confidence interval of 95% [35].

Multiple early surgical procedures supported with constant physical therapy seem to show some postnatal improvement in patients with AMC [1, 2]. Some of the surgical procedures are tendon release, repair of clubfoot, traction and casting, lengthening of the extensor, and distraction osteogenesis, which is used to improve upper airway obstruction [36, 37]. Posterior spinal instrumentation is also used to prevent angular deformities in patients with AMC and scoliosis, which makes up approximately 20% of the children with AMC [17, 37]. While most patients need multiple procedures to correct deformities and release contractures, patient‐specific therapy is also necessary for a positive outcome [37]. Moreover, the severity of the skeletal condition at birth has not shown a correlation with long‐term ambulatory status [38].

A mental outcome evaluation also bears importance for patients with AMC. It was found that about 19% of adults with AMC have some signs of depression [39]. The same study also showed that the prevalence of depression in an adult sample of individuals with AMC is similar to the rates of depression reported in the general population of the United States [39]. While age and sex were noted as independent predictors of depression, poor environmental factors and lack of occupation (work and/or volunteering) were found to be significantly related to depression symptoms in individuals with AMC [39].

The prognosis of AMC is highly variable and depends heavily on the etiology, extent of contractures, and associated abnormalities of the condition [24]. Some associative abnormalities such as brain malformations can indicate chromosomal syndromes which often result in a prognosis of restrictive cognitive outcome. (Table 1) [22] In general terms, infants with exclusive limb involvement tend to result in more favorable outcomes and a better response to non‐invasive treatment such as physical therapy, even though surgery is often needed [2]. Cases of CNS involvement or nervous system disorders, however, tend to imply a poor prognosis regarding function, autonomy, and lethality [2, 4]. Due to the variable nature of AMC prognosis, an early accurate diagnosis is necessary to allow for further testing and to provide additional counseling to the family about the future of the pregnancy [4, 24].

**TABLE 1:** Summary of genetic etiology associated with prognosis in AMC.

Genetic etiology	Prenatal diagnosis previously reported	Postnatal survival
Aneuploidy^a^	Yes	Unlikely
Copy number variant^b^	Yes	Variable
Single gene disease		
Autosomal Dominant	Yes	No
Autosomal Dominant	Yes	Yes
Autosomal Dominant	No	Yes
Autosomal Dominant	No	No
Autosomal Recessive	Yes	No
Autosomal Recessive	Yes	Yes
Autosomal Recessive	No	Yes
Autosomal Recessive	No	No
X‐linked	Yes	No
X‐linked	Yes	Yes
X‐linked	No	Yes
X‐linked	No	No
Mitochondrial/Multifactorial/Etc.		

^a^ Full extra or missing chromosome (ex Trisomy 18).

^b^ Partial chromosomal deletion or duplication.

Ultrasound scans are an exceptional tool to estimate the prognosis of AMC. Some findings like pterygia, scoliosis, and opisthotonos associated with AMC usually result in the prenatal death of the fetus [7]. Other findings like hydrops, nuchal edema, polyhydramnios, and absent gastric filling also present a high likelihood of IUFD [7]. Surviving fetuses usually do not show hydrops or nuchal edema, and polyhydramnios is rare, presenting in 25% of surviving fetuses [7]. Scoliosis and absent stomach filling have shown a strong correlation with neurological etiologies among AMC patients [7].

**FIGURE 8:**
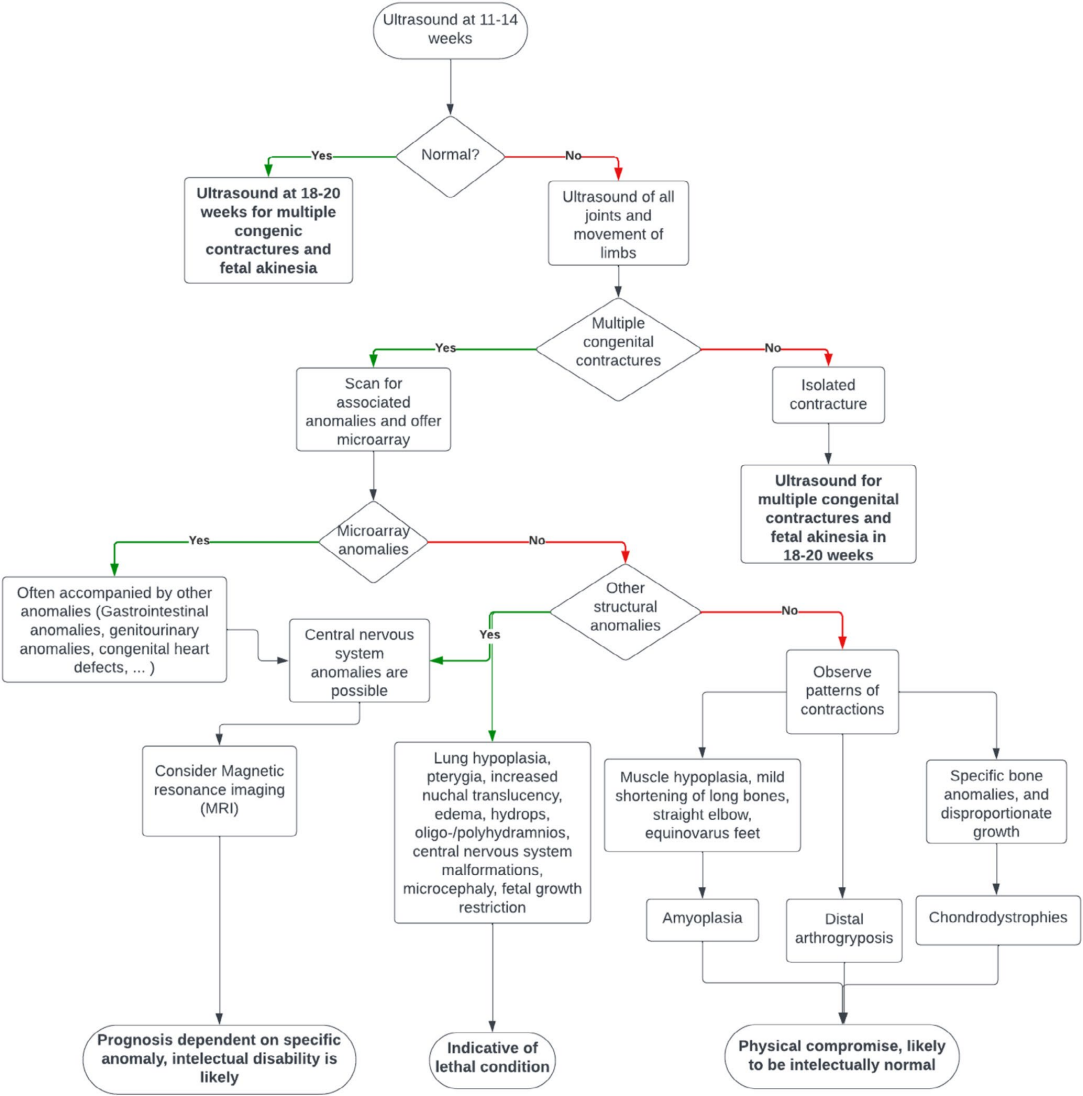
Flowchart of management and prognosis of AMC. (Adapted from Filges and Hall [23] and Adamo et al. [34]).

Due to the variable nature of the etiology of AMC, the management and prognosis of the condition can vary (Figure 8). However, in cases where multiple congenital contractures are observed, ultrasound scans should be performed at 14, 18, 20, 23, 28, and 32 weeks [40]. In the case of intrauterine fetal death or termination of pregnancy, an autopsy should be offered to understand the etiology, diagnosis, and recurrence risks [8].

## 4 Discussion

AMC is characterized by joint contractures in at least two different sites of the fetus' body [1, 2]. The condition has a large number of possible etiologies and associated abnormalities that can increase several risks for the fetus exponentially [1, 2, 6, 35]. Prognosis ranges from individuals with motile difficulties to death; therefore, early diagnosis is crucial to improve the outcome of the fetus and to provide the family with the most information so an informed decision about the pregnancy can be made [2, 4, 24].

Ultrasound is the most versatile, safe, and low‐cost tool currently available to achieve a prenatal diagnosis and estimate the prognosis of fetal AMC [41]. 3D and 4D ultrasound have shown better results at identifying fetal akinesia, but the condition is often missed by sonographers since fetal movement is not routinely scanned in prenatal exams [11, 19]. 2D ultrasound has shown at least 83% accuracy in predicting the outcome of pregnancies, and that number is enhanced when 3D and 4D ultrasound scans are added to prenatal care [7, 19]. Therefore, it is paramount in the prenatal diagnosis of the condition.

In order to improve the detection of contractures in ultrasound screenings, Filges and Hall propose the following strategies: [22, 23]

– If clubfoot, clenched hand, or any limb anomaly is detected, a careful mandatory examination of all joints and limbs should be performed, reporting the number and position in flexion, dislocation, and extension of each joint.
– Assessment of all organ systems for associated anomalies such as brain abnormalities, lung hypoplasia, hydropic signs, and webbing.
– Complementation of ultrasound with magnetic resonance imaging (MRI) should be considered where indicated;
– Repeat assessment every 2–4 weeks to improve detection of associated malformations and to evaluate the disease progression.

A standardized protocol can enhance the detection and diagnosis rates of AMC, as well as provide physicians with an optimal protocol that would promote a multidisciplinary and efficient treatment of all fetuses affected by the disease [23, 42, 43].

## 5 Conclusion

In conclusion, AMC is a rare condition characterized by contractures present in multiple joints of the fetus' body. It has a wide range of etiologies, ranging from genetic factors to intrauterine and extrauterine mechanisms. AMC has a diverse and complex prenatal diagnosis and perinatal course. Two‐dimensional ultrasound is the most commonly used tool for prenatal diagnosis of AMC, considering that the genetic cause of the condition is not yet fully understood in up to 40% of cases. 3D and 4D ultrasound, as well as fetal MRI, have proven to be useful in the prenatal diagnosis of AMC as well. Due to the variable nature of AMC, its prognosis is also variable and depends heavily on the etiology. Prenatal diagnosis of AMC allows for physicians to plan the delivery at a tertiary center that possesses the necessary equipment and staff to care for a newborn with AMC. In utero diagnosis is also useful to better inform the parents about the disease and give them an opportunity to prepare themselves to care for their child.

## Author Contributions

R.R. and M.B.L. designed the review. M.B.L., M.F.B., J.R.D.T.L., and S.E.N. performed and reviewed the literatureelectronically. M.B.L. and M.F.B. analyzed the data. S.E.N. and R.R. also revised the genetic aspects of the manuscript. All authors assisted in drafting the article, submitting it, and revising it for important intellectual content, and all authors approved the final version of the review to be published.

## Ethics Statement

The authors have nothing to report.

## Conflicts of Interest

The authors declare no conflicts of interest.

## Data Availability

Data sharing is not applicable to this article as no new data were created or analyzed in this study.
